# Appraisal of Total Phenol, Flavonoid Contents, and Antioxidant Potential of Folkloric *Lannea coromandelica* Using *In Vitro* and *In Vivo* Assays

**DOI:** 10.1155/2015/203679

**Published:** 2015-09-17

**Authors:** Tekeshwar Kumar, Vishal Jain

**Affiliations:** University Institute of Pharmacy, Pt. Ravishankar Shukla University, Raipur, Chhattisgarh 492 010, India

## Abstract

The aim of this study was to determine the impending antioxidant properties of different extracts of crude methanolic extract (CME) of leaves of *Lannea coromandelica* (*L. coromandelica*) and its two ethyl acetate (EAF) and aqueous (AqF) subfractions by employing various established *in vitro* systems and estimation of total phenolic and flavonoid content. The results showed that extract and fractions possessed strong antioxidant activity *in vitro* and among them, EAF had the strongest antioxidant activity. EAF was confirmed for its highest phenolic content, total flavonoid contents, and total antioxidant capacity. The EAF was found to show remarkable scavenging activity on 2,2-diphenylpicrylhydrazyl (DPPH) (EC_50_ 63.9 ± 0.64 *µ*g/mL), superoxide radical (EC_50_ 8.2 ± 0.12 mg/mL), and Fe^2+^ chelating activity (EC_50_ 6.2 ± 0.09 mg/mL). Based on our *in vitro* results, EAF was investigated for *in vivo* antioxidant assay. Intragastric administration of the EAF can significantly increase levels of superoxide dismutase (SOD), catalase (CAT), glutathione (GSH), and glutathione peroxidase (GSH-Px) levels, and decrease malondialdehyde (MDA) content in the liver and kidney of CCl_4_-intoxicated rats. These new evidences show that *L. coromandelica* bared antioxidant activity.

## 1. Introduction

In recent times peoples are paying their attention towards the importance of dietary antioxidant components for the betterment of human health due to the apprehension about the side effects of synthetic antioxidants. Several vegetables, herbals, and medicinal plants are playing an important role as powerful antioxidants due to the presence abundance of polyphenolic compounds [1]. Plant phenolic compounds are the products of secondary metabolism, ubiquitously present in the plant kingdom with interesting properties for animal or human health. Consumption of a variety of phenolic compounds may lower the risk of serious health disorders (chronical and degenerative diseases) due to considerable resistance to the oxidative damage caused by the ROS [2–4]. Antioxidants are micronutrients that can either directly scavenge reactive oxygen species (ROS) or prevent the generation of ROS. Overexcited antioxidant enzyme system (superoxide dismutase (SOD), catalase (CAT), and glutathione peroxidase (GPx)/oxidized glutathione reductase (GSSGRD) systems), nonenzymatic action (glutathione and vitamin E), and prooxidants are responsible for the generation of reactive oxygen and nitrogen species (ROS/RNS) such as superoxide anion (O^2−^), hydroxyl radical (HO^•^), hydrogen peroxide (H_2_O_2_), peroxyl radical (ROO^•^), singlet oxygen (O_2_), nitric oxide (NO^•^), peroxynitrite (ONOO^−^), and other free radicals [5, 6]. Various conditions like radical initiating factors and pitiable antioxidant protective system are responsible for ROS overproduction and oxidative stress.

ROS is generated inside the living system by normal aerobic respiration stimulating polymorph nuclear leukocytes and macrophages, and peroxisomes, as endogenous sources, act as second messengers and activate copious biological responses, such as release of cytokines such as interleukin-1*β* and tumor necrosis factor- (TNF-) *α* or activation of NF-*κ*B or AP-1. These released factors increase the production of ROS and further embroil the inflammatory response [7]. Several other factors like tobacco chewing, smoke, certain pollutants, organic solvents, and pesticides are the exogenous sources [8]. Generated ROS initiates the peroxidation of membrane lipids as well as a wide range of biological molecules through a process that is believed to be implicated in the etiology of several major human ailments including atherosclerotic plaque formation in artery [9], stroke, diabetes, rheumatoid arthritis, cancer, cardiovascular disease, osteoporosis, degenerative disease and senescence processes [10], causes deterioration of various food products, and finally leads to loss of nutritive value or complete spoilage [11]. For many decades, there has been a growing interest in finding novel antioxidants that safely prolong the shelf life of foods as well as combating and/or preventing ROS-mediated disorder. ROS regulates innumerable vital phenomena such as phagocytosis, regulation of cell proliferation, intracellular signaling, and synthesis of biologically active compounds and ATP [12]. Antioxidants can influence the oxidation process through simple or complex mechanisms, including prevention of chain initiation, binding of translational metal ion catalysts, decomposition of peroxides, prevention of continued hydrogen abstraction, and radical scavenging. Such natural antioxidant substances are believed to play a potential role in interfering with the oxidation process by reacting with free radicals, chelating catalytic metals, and scavenging oxygen in the biological systems [13], associated with health benefits by preventing damage due to biological degeneration, and also are effective as natural food preservatives against oxidative deterioration and microbial contamination [14, 15]. These natural antioxidants that are consumed via our diet have beneficial effects on human health, including inhibition of mutagenesis and carcinogenesis [16].

Hence there is growing interest in natural polyphenolic antioxidants, present in medicinal and dietary plants that help assuage oxidative damage [17].

*Lannea coromandelica* (*L. coromandelica*) Houtt. Merrill. (Anacardiaceae) also has a synonym* Jingini* (Sanskrit) is deciduous large tropical trees, up to 15–20 m tall, and widely distributed in Bangladesh, India, and some other tropical countries. It was commonly called as Woodier or Indian ash tree. In Ayurvedic text* Jingini* is mentioned as a substitute for* Murva* (*Marsdenia tenacissima*) [18].* L. coromandelica* showed innumerable pharmacological activities such as hypotensive [19], antimicrobial, wound healing [20], anticancerous [21], and* in vitro* antifilarial activities [22]. Plant showed the presence of various phytoconstituents like phenolic compounds, flavonoids, triterpenoids, tannins, and alkaloids [23]. Traditionally bark and leaves of* L. coromandelica* are commonly used to treat several symptoms [24–27]. The leaf juice was used to relieve ulcers and pain when taken orally. Limited studies on phenolic compounds, flavonoid content, or antioxidant activity of* L. coromandelica* have been reported so far. Therefore, the aim of the current study was to evaluate antioxidant activities of extract and fractions from* L. coromandelica* leaves using different bioassays such as DPPH radical scavenging, reducing power assay, superoxide anion radical scavenging assay, FRAP assay, ferrous ion chelating assay, cupric reducing antioxidant capacity, bleaching assay of *β*-carotene, trolox equivalent antioxidant capacity assay method, and* in vivo* antioxidant activity by determining endogenous antioxidant enzymes in carbon tetrachloride (CCl_4_) induced oxidative toxicity assay. Due to the crucial role played by phenolics and flavonoids compounds as antioxidants, the amounts of total phenolics and total flavonoids in the extract and fractions were also determined.

## 2. Materials and Methods

### 2.1. Collection of Plant Material

The plant* L. coromandelica* was collected from forest, managed by the Government of Chhattisgarh State Forest Division in November 2013. The collected plant was botanically recognized by Dr. V. P. Prasad. A voucher specimen (CNH/Tech.II/2014/70/139) was submitted to the Central National Herbarium, Howrah, India. The fresh leaves were carefully washed with deionized water and dried at room temperature. The dried leaves were manually ground into fine powder.

### 2.2. Preparation of Extract

Air dried leaf powder (8 g) of* L. coromandelica* was extracted with 80% methanol by Buchi Speed Extractor (pressure 100 bar, temperature 100°C, heat-up time 5 min, hold time 3 min, number of cycles 5, and time to cycle end 5 min), and extraction was repeated three times. The extract was mixed and dried to obtain crude methanolic extract (CME). Further CME of leaf of* L. coromandelica* (CME) was fractionated with ethyl acetate and water. The CME and its fractions (ethyl acetate fraction (EAF) and aqueous fraction (AqF)) were filtered through filter paper Whatman number 1. Then solvents were removed by using rotary evaporator (Ika RV 10). The yield of extract and its fractions was measured and maintained at 4°C for further use.

### 2.3. Chemicals

DPPH (1,1-diphenyl-2-picrylhydrazyl), gallic acid, ascorbic acid, quercetin, butylated hydroxytoluene (BHT), Folin-Ciocalteu's phenol reagent, potassium ferricyanide, potassium acetate, trichloroacetic acid, ammonium molybdate, aluminum chloride hexahydrate, sodium carbonate, sodium phosphate monobasic, nicotinamide adenine dinucleotide (NADH), nitroblue tetrazolium (NBT), phenazine methosulphate (PMS), ethylene diamine-tetra-acetic acid (EDTA), ABTS [2,2′-azino-bis(3-ethylbenzothiazoline-6-sulfonic acid)], TPTZ [2,4,6-tris(2-pyridyl)-1,3,5-triazine], 3-(2-pyridyl)-5,6-bis(4-phenyl-sulfonic acid)1,2,4-triazine (ferrozine), ferric chloride, trolox, *β*-carotene, and Tween 40 were purchased from Sigma-Aldrich Chemie (Steinheim, Germany). All other solvents and chemicals were of analytical grade and obtained from Merck, Mumbai (India).

### 2.4. Preliminary Phytochemical Screening (Qualitative Analysis)

The preliminary phytochemical studies were performed for testing the different chemical groups present in CME, EAF, and AqF [28].

### 2.5. Phytochemical Investigation

#### 2.5.1. Determination of Total Phenolic Compounds

Total phenolic contents were assessed through the method described by Guo et al. [29] with some minor modifications. The sample mixture contains an aliquot (1 mL) of appropriately diluted extracts (3 mg/mL) or standard solutions of gallic acid which was added to 10 mL volumetric flask containing 8 mL of ddH_2_O. Now add 1 mL of Folin and Ciocalteu's phenol reagent to the above mixture and shake it. After 3 min, 1 mL of 35% Na_2_CO_3_ solution was added with mixing to reach the reaction system 10 mL. The reaction mixture was mixed thoroughly and allowed to stand for 90 min at 23°C in the dark. Absorbance of all the sample solutions against a blank was measured at 725 nm using the UV-spectrophotometer (Shimadzu-1800). Calibration curve was constructed with different concentrations of gallic acid (2–12 *μ*g/mL) as the standard and ddH_2_O was used as reagent blank. The results were expressed as mg gallic acid equivalents (GAE)/g extract. All samples were analyzed in three replications.

#### 2.5.2. Determination of Total Antioxidant Capacity (TAC)

The total antioxidant capacities of samples were evaluated by using the phosphomolybdenum method [30]. The assay is based on the reduction of Mo (VI) to Mo (V) by the antioxidant compounds and subsequent formation of a green phosphate/Mo (V) complex at acidic pH. 300 *μ*L of samples were mixed with 3 mL of reagent solution consisting of 28 mM sodium phosphate monobasic, 4 mM ammonium molybdate, and 0.6 M sulfuric acid. The mixture was incubated at 95°C for 90 min, and later the absorbance was taken at 695 nm. The total antioxidant capacities were expressed as mg ascorbic acid equivalent per g dry extract.

#### 2.5.3. Determination of Total Flavonoid Content (TFC)

Total flavonoid content was determined by the method described by Lin and Tang [31]. According to this method aliquots were prepared by dissolving 0.1 g of extracts in 1 mL deionized water. 0.5 mL of sample solution was taken out and was mixed with 1.5 mL of 95% alcohol, 0.1 mL of 10% aluminum chloride hexahydrate (AlCl_3_·6H_2_O), 0.1 mL of 1 M potassium acetate (CH_3_COOK), and 2.8 mL of deionized water. The mixture was kept for incubation at room temperature for 40 min. After the reaction time absorbance was measured at 415 nm against a deionized water blank on spectrophotometer (Shimadzu-1800). Quercetin was chosen as a standard (0–50 mg/L). Total flavonoids were expressed as mg quercetin equivalents (QE)/g dry matter from herbs. Absorbance was determined in triplicate for all extracts, respectively.

#### 2.5.4. HPLC Analysis

*(1) HPLC Conditions*. HPLC analysis was carried out using autosampler Water 2459 series. Separation was achieved at 25°C on a 250 mm × 4.6 mm i.d. 5 *μ*m, C18 column (Water SunFire Column). The mobile phase consisted of methanol and 2% aqueous acetic acid; gradient mode (0–15 min, 40–60% v/v methanol and 15–20 min, 60% v/v methanol) was pumped using pump with a flow rate of 1 mL/min. Data analysis was performed using Water Empower software. Standard curve and retention times were calibrated using pure standards compounds (gallic acid, catechin, ellagic acid, and quercetin) (Sigma-Aldrich Co., St. Louis, MO, USA). The result was expressed as milligram per gram (mg/g) of extract.

*(2) Preparation of Sample Solution*. Leaves of* L. coromandelica* (10 g) were extracted by hydrolysis of sample with 30 mL of methanol, ethyl acetate, and water separately under refluxed for 1 hour. Extract obtained was filtered using Whatman filter paper number 42. 10 mL of distilled water was added to the filtrate and evaporated to a volume of 10 mL. Samples were analyzed immediately after extraction in order to avoid possible chemical degradation [32].

### 2.6. Antioxidant Activities (AOA) Measurement

The antioxidant activities (AOA) of CME and its fractions (EAF and AqF) of* L. coromandelica* were gauged using the following methods.

#### 2.6.1. Measurements of the DPPH Radical Scavenging Activity

DPPH radical-scavenging activities of the CME and fractions were measured according to the method described by Myagmar and Aniya [33]. DPPH is a stable free radical at room temperature and accepts an electron or hydrogen radical to become stable diamagnetic molecule. In scavenging activity the reaction mixture contains 1.0 mL of 0.1 mM DPPH-ethanol solution, 0.95 mL of 0.05 M Tris-HCl buffer (pH 7.4), and 50 *μ*L of the different concentrations of extract and fractions as well as for standards (0–100 *μ*g/mL). After 30 sec the absorbance was measured in spectrophotometer (Shimadzu-1800) at 517 nm and the free radical scavenging activity was calculated in the form of EC_50_ value according to the standard equation using ascorbic acid as control. Lower value of absorbance indicates the higher free radical scavenging activity of the reaction mixture. The reduction capability of DPPH radicals was determined by decreased absorbance, which is induced by antioxidants. The EC_50_ value is the concentration of antioxidant required for 50% scavenging of DPPH radicals in the quantified time period. All determinations were performed in triplicate. In the blank control, the sample was substituted with deionized water. In the positive control, the sample was substituted with BHT (butylated hydroxytoluene):

(1) $$\begin{array}{c} \text{DPPH scavenged }\left(\;\%\;\right)=\left[\;\left(\;1-\frac{A_{\text{test }517}}{A_{\text{control }517}}\;\right)\;\right]\times 100. \end{array}$$

EC_50_ value is the concentration of antioxidant required for 50% scavenging of DPPH radicals in the specified time period. All determinations were performed in triplicate.

#### 2.6.2. Reducing Power Assay

The reducing power of extracts was determined according to the method reported by Shen et al. [34]. 1.0 mL of sample (0–30 mg/mL) in 0.02 M PBS buffer (pH = 7.4) was mixed with 1.0 mL potassium ferricyanide (1.0%, w/v). After this, the mixture was incubated at 50°C for 20 min. Then 1.0 mL of trichloro acetic acid (10.0%, w/v) was added to the mixture to terminate the reaction. After that, the solution was mixed with 0.4 mL ferric chloride (0.1% w/v) for 10 min. Ascorbic acid (0–5 mg/mL) was used as standard. The absorbance was measured at 700 nm. Increased absorbance of the reaction mixture indicated increased reducing power.

#### 2.6.3. Superoxide Anion Radical-Scavenging Assay

The superoxide scavenging ability of the extract and its fractions was measured by the nitroblue tetrazolium (NBT) reduction method [35]. According to this method anion (O^2−^) was generated by reduction of yellow dye (NBT^2+^) to produce the blue formazan by xanthine oxidase,* in vitro*. According to this method stock solution of sample to a concentration of 100 mg/mL was prepared. Different amounts of stock solution were taken and the final volume was made up to 400 *μ*L using phosphate buffer (0.1 M, pH 7.4). The reaction mixture containing 400 *μ*L of different serial dilutions, 1 mL of PMS (60 *μ*M), 1 mL of NADH (677 *μ*M), and 100 *μ*L of NBT (144 *μ*M) in phosphate buffer (0.1 M, pH 7.4), was incubated at room temperature for 5 min. The intensity of color was measured using spectrophotometer at 560 nm against a blank. Samples and positive controls were added to the reaction mixture, in which O^2−^ was scavenged, thereby inhibiting the NBT reduction. All the measurements were taken in triplicate and the mean values were calculated. Percentage of SRSA was calculated using the following equation:

(2) $$\begin{array}{c} \%\text{ SRSA}=\left[\;1-\frac{{\text{Abs}}_{\text{sample 560}}}{{\text{Abs}}_{\text{control 560}}}\;\right]\times 100. \end{array}$$

#### 2.6.4. Ferric Reducing Antioxidant Power (FRAP) Assay

The ferric reducing antioxidant power assay of extract was measured according to the method of Benzie and Strain [36] with some minor modifications. The stock solutions included 300 mM acetate buffer (pH 3.6), 10 mM TPTZ (2,4,6-tri(2-pyridyl)-s-triazine solution in 40 mM hydrochloric acid), and 20 mM ferric chloride hexahydrate solution. The fresh working solution was prepared by mixing 25 mL acetate buffer, 2.5 mL TPTZ solution, and 2.5 mL ferric chloride hexahydrate solution, which was then warmed to 37°C before use. 100 *μ*L of each of crude extract and fractions (1 mg/mL) was taken in separate test tubes and 2900 *μ*L of FRAP solution was added to each to make a total volume of 3 mL. The plant crude extracts were allowed to react with the FRAP solution in the dark for 30 min. The absorbance of the coloured product (ferrous tripyridyltriazine complex) was measured at 593 nm. Additional dilution was needed if the FRAP value measured was over the linear range of the standard curve (200–1000 *μ*M FeSO_4_). The results are expressed in *μ*mol TE/mL. At low pH, when a ferric-tripyridyltriazine (Fe III-TPTZ) complex is reduced to the ferrous (Fe II) form, an intense blue colour develops with an absorption maximum at 593 nm.

#### 2.6.5. Ferrous Ion-Chelating Activity

Ferrous ion-chelating activity of the extracts was measured by the method described by Guo et al. [37]. One milliliter of each sample was mixed with 50 *μ*L of 2 mM FeCl_2_·4H_2_O and 3.7 mL of Milli-Q water. After the addition of 5 mM ferrozine (200 *μ*L), the mixture was allowed to incubate for 10 min at room temperature. Later, the absorbance was read at 562 nm. The activity was calculated by using the same formula which was used for the DPPH radical scavenging activity. EDTA was used as a reference compound.

#### 2.6.6. Cupric Reducing Antioxidant Capacity (CUPRAC)

The cupric ion reducing capacities of the extracts were determined as per Apak et al. [38] with slight modifications. One milliliter of each of 10 mM CuSO_4 _(Cu II), 5 mM neocuproine, and 1 M ammonium acetate buffer (pH 7.0) solutions was added to a test tube. After that, 0.5 mL of sample solutions was added, and the total volume was completed to 4.1 mL with water. After 1 h of incubation period at room temperature, the absorbance was recorded at 450 nm. Ascorbic acid was used as standard, and the results were expressed as mg ascorbic acid equivalent/g dried extract.

#### 2.6.7. Bleaching Assay of *β*-Carotene in a Linoleic Acid System

The antioxidant activity (AOA) of the different extract/fractions was evaluated using the *β*-carotene-linoleic acid assay followed by the method of Amarowicz et al. [39]. In brief, a solution of *β*-carotene was prepared by dissolving 2 mg of *β*-carotene in 10 mL of chloroform. 2 mL of this solution was pipetted into a 100 mL round-bottom flask. After chloroform was removed under vacuum, using a rotary evaporator at 40°C, 40 mg of purified linoleic acid, 400 mg of Tween 40 as an emulsifier, and 100 mL of aerated distilled water were added to the flask with vigorous shaking. Aliquots (4.8 mL) of this emulsion were transferred into a series of tubes containing 200 *μ*L of the extract (200 ppm in methanol). The total volume of the systems was adjusted to 5 mL with methanol. As soon as the emulsion was added to each tube, the zero-time absorbance was measured at 470 nm with a spectrophotometer (Shimadzu-1800). Subsequent absorbance readings were recorded over a 2 h period at 20 min intervals by keeping the samples in a water bath at 50°C. Blank samples, devoid of *β*-carotene, were prepared for background subtraction.

#### 2.6.8. Trolox Equivalent Antioxidant Capacity (TEAC)/ABTS^•+^ Radical-Scavenging Assay

This assay was carried out according to the procedure as previously described by Re et al. [40]. 5 mL of 7 mM aqueous ABTS (2-2′-azinobis-(3-ethylbenzothiazoline-6-sulfonate)) is mixed with 2.45 mM potassium persulfate (1.6 *μ*L) and the mixture is kept in the dark at room temperature for 16 h to generate ABTS^•+^ radical cation (blue-green colour). Later, ABTS^•+^ solution was diluted with ethanol to achieve an absorbance of 0.70 ± 0.025 at 750 nm, the wavelength of maximum absorbance in the visible region. Sample solutions were prepared by dissolving 1 mg of extract in 5 mL of 70% aqueous ethanol. Then, 2 mL ABTS^•+^ solution was added to 20 *μ*L of different concentrations (10–500 *μ*g/mL) of extracts. These samples were vortexed and incubated in the dark for 6 min. The absorbance was measured at 750 nm. Absorbance of control (3.0 mL ABTS^•+^ solution with 30 *μ*L water) was recorded in advance (*A*_control_). The percentage of absorbance inhibition was calculated using the formula

(3) ABTS radical-scavenging activity%=1−AB×100,

where  *A* is absorbance value of testing solution and *B* is absorbance value of control solution.

A standard curve was obtained by using trolox standard solution at various concentrations (ranging from 0 to 15 *μ*M final concentration) in ethanol. The antioxidant activity was reported as mg of trolox equivalent to the antioxidant activity (T)/g dry sample (d.s.) or mg of ascorbic acid equivalent to the antioxidant activity (AA)/g d.s. All determinations were carried out three times.

### 2.7. *In Vivo* Antioxidant Activity

#### 2.7.1. Animals

Wistar rats (150–250 g) of both sexes were used for antioxidant activity. The animals were obtained from animal house of Rungta College of Pharmaceutical Sciences and Research, Bhilai, Chhattisgarh, India. The animals were acclimatized in the pharmacology research laboratory, for seven days before the start of experiments. The animals were kept in groups of six per cage under standard environmental conditions (12 : 12 h light : dark cycle at 25 ± 2°C) and were fed ad libitum on a diet of standard pellets and water. Food was withdrawn 12 hours before experiments. The studies were approved by the Institutional Animal Ethics Committee (1189/PO/a/08/CPCSEA) and were performed as per the Guidelines of Committee for the Purpose of Control and Supervision of Experiments on Animals (CPCSEA), Chennai, India.

#### 2.7.2. Acute Toxicity Study

Acute toxicity study was carried out as per the OECD Guidelines 420. The animals were administered orally with different doses of extract. The animals were continuously observed for the autonomic and behavioral changes for 12 hrs and the mortality was observed for 24 hours. No mortality was found up to a dose of 2000 mg/kg which was taken as the end point dose till the completion of 24 hours. The doses of 100 mg/kg, 200 mg/kg, and 400 mg/kg were selected for the further activity on the basis of pilot studies performed at our lab (data not shown).

#### 2.7.3. Carbon Tetrachloride (CCl_4_) Induced Oxidative Toxicity

Rats were divided into six groups (*n* = 6 in each group). Group I received vehicle only (0.3% CMC-Na; 1 mL/kg body weight, p.o.) for 5 days and olive oil (1 mL/kg body weight, i.p.) on days 2 and 3. Group II (CCl_4_) received 0.3% CMC-Na (1 mL/kg body weight, p.o.) for 5 days and mixture of CCl_4_ and olive oil (1 : 1 : 2 mL/kg body weight, i.p.) on days 2 and 3. Groups III to VI were treated with the standard drug vitamin E (50 mg/kg body weight, p.o.) and ethyl acetate extract of* L. coromandelica* (100, 200, and 400 mg/kg body weight, p.o.), respectively, for 5 days. Additionally, they received a dose of 1 : 1 CCl_4_ and olive oil (2 mL/kg bodyweight, i.p.) on days 2 and 3, 30 min after the administration of the standard or extracts. On day 6, animals were sacrificed by decapitation and liver was carefully dissected and cleaned of extraneous tissue. 10.0% w/v homogenates were prepared by mincing and homogenizing 1 g of liver with cold 50 mM potassium phosphate buffer (pH 7.4) and centrifuged at 6000 rpm for 10 min at 4°C. The cell-free supernatant was used for the biochemical testing.

#### 2.7.4. Biochemical Determinations

Antioxidant enzymes activities were determined in liver and kidney homogenates for maleic dialdehyde (MDA), superoxide dismutase (SOD), catalase (CAT), glutathione (GSH), and glutathione peroxidase (GSH-px). The superoxide dismutase activity was measured according to the previously described method by Ilouno et al. [41]. This method was evaluated on the basis of inhibition of the reduction of nitroblue tetrazolium (NBT) of the homogenates at wave length 560 nm. Briefly, homogenate (10 *μ*L) was added with 0.1 mol/L EDTA, 1.5 mmol/L NBT, 1.5 mg sodium cyanide/100 mL, 0.12 mmol/L riboflavin, and 0.067 mol/L phosphate buffer (pH 7.8) at final volume of 300 *μ*L. Reduced NBT was quantified at 560 nm using spectroscopic. SOD activity was expressed as U/mg protein. Catalase activity was assayed by the method of Aebi [42]. Activity of catalase was based on the rate of hydrogen peroxide (H_2_O_2_) reduction in reaction mixture of 50 mM phosphate buffer (pH 7.0), 10 mM H_2_O_2_, and homogenates. The reduction rate of H_2_O_2_ was followed for 30 s at room temperature and absorbance was measured at 240 nm. CAT activity was expressed as U/mg protein. The MDA levels were determined using the 2-thiobarbituric acid (TBA) as previously method described by Jamall and Smith [43]. Reaction mixture of 200 *μ*L of homogenates/standard, 200 *μ*L of sodium dodecyl sulfate, 1500 *μ*L of acetic acid solution and 1500 *μ*L of TBA (thiobarbituric acid), and 600 *μ*L of Milli-Q water were kept at 95°C for 1 h. After cooling, 2000 *μ*L of the reaction mixture was mixed with 2000 *μ*L of TCA (trichloroacetic acid) and centrifuged at 1000 rpm for 10 min. The absorbance of the supernatant was observed at 532 nm. The results were expressed as nmol/mg protein. 1,1,3,3-Tetraethoxypropane (TEP) was used as a standard. Glutathione peroxidase (GSH-Px) activity was measured by oxidation of glutathione by the enzyme described by Rotruck et al. [44] with minor alteration. In this method, the reaction mixture (850 *μ*L) contained 200 *μ*L of 0.4 mol/L phosphate buffer (pH 7.0), 200 *μ*L of 0.4 mmol/L EDTA, 100 *μ*L of 10 mmol/L sodium azide and 100 *μ*L of H_2_O_2_, and 200 *μ*L of glutathione. 150 *μ*L of homogenates was added to the above mixture and was incubated for 10 min at room temperature. The activity of enzyme was read at 420 nm. The determination of reduced glutathione (GSH) in liver and kidney tissues was according to the method of Moron et al. [45]. In this method, 1.0 mL of homogenate was precipitated with 1.0 mL of trichloroacetic acid (TCA) and the precipitate was removed by centrifugation. To 0.5 mL of supernatant, 2.0 mL of 5,5′-dithiobis(2-nitrobenzoic acid) (DTNB or Ellman's reagent) was added and the total volume has reached to 3.0 mL with phosphate buffer (pH 7.0). Reduction of DTNB by sulfhydryl groups to 2-nitro-5-mercaptobenzoic acid (intense yellow color) was measured at 412 nm. The results were expressed in mg per g protein (mg/g protein).

### 2.8. Statistical Analysis

The results were presented as a mean ± SD of the indicated number of experiments (*n* ≥ 3). One-way analysis of variance (ANOVA) followed by Dunnett's test was used to find the statistical differences between groups. All statistical analyses were performed using GraphPad Prisms (product version 5.03).

## 3. Results and Discussion

### 3.1. Preliminary Phytochemical Screening

Preliminary phytochemical screening of extract and fractions of* L. coromandelica* showed the presence of alkaloids, steroid, triterpenes, flavonoid, and polyphenolics compounds (Table 1).

### 3.2. Phytochemical Investigation

#### 3.2.1. Amount of Extractable Compounds versus Extractable Phenolic Compounds

The result of ability of different solvents used for the extraction/fractionation of phenolic compounds is given in Table 2. In general, the amount of total extractable compounds decreased with decreasing polarity of the solvent in the order water, methanol, and ethyl acetate [46]. With this solvent, the highest amount of TEC was found to be 798.5 ± 0.19 mg/g in AqF followed by CME 342 ± 0.21 mg/g and EAF 256.7 ± 0.27 mg/g. These findings are in good agreement with the fact that the organic solvents are the poor solvents for the extraction of polyphenol without added water [47].

#### 3.2.2. Total Phenolic Content

The total polyphenol content of methanol extract and different fractions prepared by partitioning of methanol extract with different solvent polarities was determined from regression equation of calibration curve and expressed in gallic acid equivalents between 15.71 ± 0.17 and 48.18 ± 0.18 mg GAE/g of extract/fraction as shown in Table 2. It was noticed that the EAF shows higher total poly phenolic contents than CME followed by AqF. Phenolic compounds react with reactive oxygen species and have the ideal chemical properties for antioxidant activity since they act both as hydrogen and electron donors. It can also chelate prooxidant metals and modify the activity of enzymes [48]. Low-molecular-weight phenolic compounds and high-molecular-weight polyphenols can be readily extractable using ethyl acetate an extraction solvent with a higher degree of selectivity [49].

Now, it becomes clear that the there was no significant correlation between the amount of total extractable compounds and the total phenolic compounds. Hence the amount of total phenolic compounds is influenced by extraction solvents.

#### 3.2.3. Total Antioxidant Capacity (TOAC)

Total antioxidant capacity test (TOAC) is based on the reduction of Mo^6+^ to Mo^5+^ by the extracts and subsequent formation of a green phosphate/Mo(V) complex at acidic pH with a maximal absorption at 695 nm [50]. Global antioxidant activity of* L. coromandelica* extract/fractions was expressed as mg ascorbic acid equivalents (AAE)/g extract between 30.4 ± 0.09 and 88.6 ± 0.04 mg AAE/g. The results for total antioxidant capacity are given in Table 2.

According to our literature survey, this is the first report on total antioxidant capacity of the leaf of* L. coromandelica* by the phosphomolybdenum complex due to presence of various phytochemicals, specially phenolic and flavonoidal compounds [51]. Current studies have shown that many polyphenols and flavonoids contribute significantly to the total antioxidant activity obtained from plant [52], byproduct [53], and several halophytes [54].

#### 3.2.4. Total Flavonoid Content

The total flavonoid content of methanol extract and different fractions determined as quercetin equivalents is presented in Table 2. The greatest quantity of flavonoid content was found in EAF (41.4 ± 0.12 mg QE/g d.m.), followed by CME (29.1 ± 0.08 mg QE/g d.m.) and AqF (13.8 ± 0.24 mg QE/g d.m.).

#### 3.2.5. HPLC Analysis

**Figure 1:**
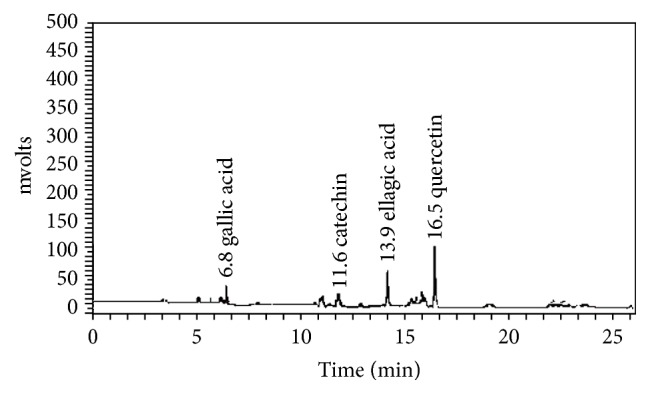
HPLC chromatogram of* L. coromandelica*.

It has been reported that the antioxidant compounds found in* L. coromandelica* leaf extract were phenolic compound (gallic acid, catechin, and ellagic acid) and flavonol (quercetin) using gradient reversed-phase HPLC system [55]. Both compounds have good absorption at 254 nm; this wavelength was used for analysis. This method was employed to separate, identify, and quantify the phytoconstituents in the extract. The concentration was determined by calculating the HPLC peak areas which are proportional to the amount of analytes in peak and presented as the mean of the two determinations which was highly repeatable. The result indicated that a mixture of methanol and 2% aqueous acetic acid, gradient mode (0–15 min, 40–60% v/v methanol, and 15–20 min, 60% v/v methanol) could separate all components in less than 25 min with agreeable peak resolution. Gallic acid, catechin, ellagic acid, and quercetin were eluted with the retention time of 6.8, 11.6, 13.9, and 16.5 min, respectively (Figure 1) and low quantity of ellagic acid (1.83 mg/g extract, 1.28 mg/g extract, and 1.06 mg/g extract) and quercetin (1.09 mg/g extract, 0.91 mg/g extract, and 0.64 mg/g extract) was detected in EAF, CME, and AqF, respectively. Gallic acid and catechin are found in very small quantity (not mentioned here). This HPLC method is simple and rapid. In addition, ellagic acid and quercetin should be considered as an indicative marker for the standardization of* L. coromandelica*.

### 3.3. *In Vitro* Antioxidant Activities (AOA) Measurement

#### 3.3.1. DPPH Radical Scavenging Activity

DPPH^•^ is a free radical with a purple or deep-violet color and accepts an electron or hydrogen radical to become a stable diamagnetic molecule with a yellow color (DPPH) by reacting with natural and synthetic antioxidants. The reduction capability of DPPH^•^ depends on the hydrogen donating ability of the antioxidant and was determined by the decrease in absorbance induced by antioxidants [56]:

(4) $$\begin{array}{c} {\text{DPPH}}^{•}+\text{AH}=\text{DPPH}+\text{H}+{\text{A}}^{•} \end{array}$$

This radical species provides an easy and simple method to evaluate antioxidant potential of all antioxidants. Radical scavenging activity of extract and fractions against stable DPPH^•^ was determined spectrophotometrically at 515 nm. This assay illustrates a decrease in the concentration of DPPH radical due to the scavenging ability of the soluble phytoconstituents present in extract and fractions. Decrease the concentration of DPPH radical due to the scavenging ability of each concentration of both standards and plants extracts. Ethyl acetate fraction has shown stronger DPPH scavenging activity rather than methanolic extract and water fractions. The DPPH scavenging effect of both standards and plants extracts on the DPPH radical decreased in the order of BHT > EAF> CME > AqF and was 91.9%, 71.4%, 56.2%, and 42.2% at the concentration of 100 *μ*g/mL, respectively. The extract/fractions were assayed over a range of dilutions to establish the concentration of each extract/fraction for determination of EC_50_ value. Lower EC_50_ value reflects better DPPH radical scavenging activity. EC_50 _of BHT was found to be 46.4 ± 0.23 *μ*g/mL whereas EAF (63.9 ± 0.64 *μ*g/mL) was the most active scavenger of DPPH radical than CME (EC_50_ = 86.5 ± 0.78 *μ*g/mL). AqF did not show the significant activity for scavenging DPPH radicals (Table 3). Only CME and EAF were found to be capable of scavenging DPPH radicals in a concentration-dependent manner. It has been found that many antioxidant molecules such as ascorbic acid, flavonoids, BHT, tocopherol, and tannins reduce and decolorize DPPH due to their electron accepting capacity [57].

#### 3.3.2. Reducing Power Assay

Reducing power assay is often used to evaluate the ability of antioxidant to donate electron. Many reports have revealed that there is a direct correlation between antioxidant activities and reducing power of certain plant extracts was associated with their antioxidant activity [37]. In the reducing power assay, the presence of antioxidants reductants in the extracts causes reduction of the Fe^3+^/ferricyanide complex to its ferrous form and is monitored by measuring the formation of Perl's Prussian blue of ferrous form at a wavelength of 700 nm for the extracts ranging in concentration from 0 to 30 mg/mL. The reducing power of extract and fractions of leaf was compared with ascorbic acid as standards. All three samples showed reducing power capacity in a concentration-dependent manner, but less than standard. The reducing power of all samples increased with the concentrations. The reducing power of CME, EAF, and AqF was 1.23 ± 0.05, 0.91 ± 0.12, and 0.57 ± 0.23 at 30 mg/mL, respectively. However, ascorbic acid showed excellent reducing power of 1.72 ± 0.08 at 5 mg/mL, which is highly significantly than that of CME, EAF, and AqF (*P* < 0.05). Interesting fact about the EC_50 _values is that the reducing power of EAF is lesser than the CME but showed higher antioxidant activity. The aqueous extract had the lowest reducing power. EC_50_ values of the extract and fractions in reducing power were significantly different (*P* < 0.05) from the EC_50_ values (Table 3) obtained for ascorbic acid (1.3 ± 0.04 mg/mL) > EAF (8.2 ± 0.12 mg/mL) > CME (17.1 ± 0.24 mg/mL) > AqF (23.6 ± 1.2 mg/mL). According to the results in the present study, it is suggested that CME has a remarkable potency to donate electron to reactive free radicals, converting them into more stable nonreactive species and terminating the free radical chain reaction.

#### 3.3.3. Superoxide Anion Radical-Scavenging Assay

Under the various oxidative stress conditions, the concentration of oxidative species can increase dramatically in all cells which persuade oxidative damage in biomolecules. The superoxide anion is the most common free radical generated* in vivo* by several oxidative enzymes, including xanthine oxidase (XO), which converts hypoxanthine to xanthine and consequently uric acid. Approximately 1–3% of the oxygen is converted to superoxide radical anion (O^2−^). Superoxide dismutase (SOD) is the cellular antioxidant enzyme that can dismutate O^2−^ *in vivo*.* In vitro* generation of superoxide anions by PMS/NADH system and could be easily monitored by the reduction of NBT. Extract and fractions of the* L. coromandelica* exhibited dose-dependent superoxide radical-scavenging activities. When at 10 mg/mL, superoxide anion radical-scavenging activities were in the following order: BHT (88.7%) > EAF (58.4%) > CME (36.4%) > AqF (31.2%). At 30 mg/mL, the order is EAF (70.4%) > CME (60.8%) > AqF (50.4%). EC_50_ values of the extract and fractions in superoxide anion radical scavenging activity were BHT (4.12 ± 0.18 mg/mL) > EAF (11.9 ± 0.23 mg/mL) > CME (18.72 ± 0.67 mg/mL) > AqF (23.89 ± 0.92 mg/mL) having significantly result (*P* < 0.05) (Table 3). Hence it could be seen that EAF might exhibit a better scavenging of superoxide radicals than other extracts/fractions.

#### 3.3.4. Ferric Reducing Antioxidant Power (FRAP) Assay

Fe^3+^ reduction is an important aspect regarding electron donation as well as being one of the major mechanisms of actions of antioxidant activity. FRAP test is frequently used to measure the antioxidant capacity [12]. The principle of this method is based on the reduction of a ferric 2,4,6-tris(2-pyridyl)-1,3,5-triazine (Fe^3+^-TPTZ) to ferrous, coloured form (Fe^2+^-TPTZ); an intense blue color is developed in the presence of extract, fractions, and compounds [58]. FRAP assay showed positive correlation between reducing power and phenolic content in* L. coromandelica* extract and fractions. FRAP values for EAF, CME, and AqF were 187.4 ± 1.45, 112.2 ± 2.12, and 74.5 ± 2.85 equivalent *μ*mol TE mL^−1^, respectively. Result shows that all the phenolic compounds act as reducing agents, hydrogen donators, and singlet oxygen quenchers (redox properties) [59].

#### 3.3.5. Ferrous Ion-Chelating Activity

Iron can initiate the lipid peroxidation by Haber-Weiss and Fenton-type reactions in a biological system and also accelerates peroxidation by decomposing lipid hydroperoxides into peroxyl and alkoxyl radicals that can themselves abstract hydrogen and initiate the chain reaction of lipid peroxidation. Ferrous ions are generally present in food systems and are considered as effective prooxidants. Ferrozine and Fe^2+^ form coloured complex (violet colour). In the presence of chelating agents, ferrozine-Fe^2+^ ion coloured complex is disturbed, resulting in a decrease in colour of the complex [60]. Measurement of the colour reduction allowed to estimate the metal-chelating activity for the coexisting chelator. EDTA, a well-known chelating agent, was used as reference compound [61]. Chelating effect of extract and fractions was compared with EDTA as standard on ferrous ions in a dose-dependent manner. EAF, CME, and AqF chelated ferrous ions by 76.8 ± 1.83% at 20 mg/mL, 62.1 ± 0.12% at 30 mg/mL, and 51.3 ± 0.56% at 40 mg/mL, respectively. EDTA, used as a positive control, showed excellent chelating ability of 99.9 ± 0.43% at a concentration as low as 0.29 mg/mL. From the estimated EC_50_ values, defined as the concentration of extract required to chelate 50% of the available iron (II), it can be seen that the most effective iron (II) chelating extract was EAF (6.2 ± 0.09 mg/mL) followed by CME (16.2 ± 0.25 mg/mL) and AqF (35.8 ± 0.57 mg/mL), in decreasing order. These were significantly (*P* < 0.05) different in efficacy from the EDTA (0.23 ± 0.05 mg/mL) (Table 3).

#### 3.3.6. Cupric Reducing Antioxidant Capacity (CUPRAC)

Chromogenic oxidizing agent (copper(II) neocuproine [Cu(II)-Nc]) is presently widely used for measurements of antioxidant capacity index for dietary polyphenols, flavonoids, and vitamins C and E. The method is based on the measurement of the developed absorbance at 450 nm of a stable copper(I) neocuproine complex [62]. In the presence of antioxidant molecules copper(II) was reduced to copper(I). This method should be advantageous over ferric reducing antioxidant power (FRAP) because the redox chemistry of copper(II) as opposed to that of ferric ion should involve faster kinetics [63]. The results of CUPRAC were 67.2 ± 0.09, 24.4 ± 0.16, and 14.3 ± 0.21 for EAF, CME, and AqF, respectively. The value of CUPRAC was expressed as mg ascorbic acid equivalents (AAE)/g extract. According to the results, EAF had the highest cupric reducing power while AqF had the least.

#### 3.3.7. Bleaching Assay of *β*-Carotene in a Linoleic Acid System

In this system, the peroxyl free radicals were generated due to oxidation of linoleic acid by abstraction of hydrogen atom from diallylic methylene groups of linoleic acid located on carbon-11 between two double bonds. The generated peroxyl radicals decolorize the highly unsaturated *β*-carotene (orange colour) in the absence of antioxidant [64]. Antioxidant compounds present in the extract/fractions minimize or prevent the oxidation of *β*-carotene by hydroperoxides radicals and can be measured spectrophotometrically [65]. Hydroperoxides formed in this system will be degraded by the antioxidants from the extract/fractions. Hence the degradation rate of *β*-carotene solely depends on the antioxidant activity of the extract/fractions. Most studies showed there was no correlation between total phenolic contents and bleaching capacity of *β*-carotene [66]. It was clear that the presence of antioxidants in the leaf extracts/fractions reduced the oxidation of *β*-carotene by hydroperoxides from these extracts/fractions. There were significant differences (*P* < 0.05) between the extract/fractions, control, and BHA effect. EAF and CME were better in their effect on reducing the oxidation of *β*-carotene than AqF fraction and that their degradation rate of *β*-carotene clearly depends on their antioxidant activity. There was a correlation between the degradation rate and the bleaching of *β*-carotene, where the extract with the lowest *β*-carotene degradation rate exhibited the highest antioxidant activity. All extracts had a lower antioxidant activity than BHA.

#### 3.3.8. Trolox Equivalent Antioxidant Capacity (TEAC)/ABTS^•+^ Radical-Scavenging Assay

**Figure 2:**
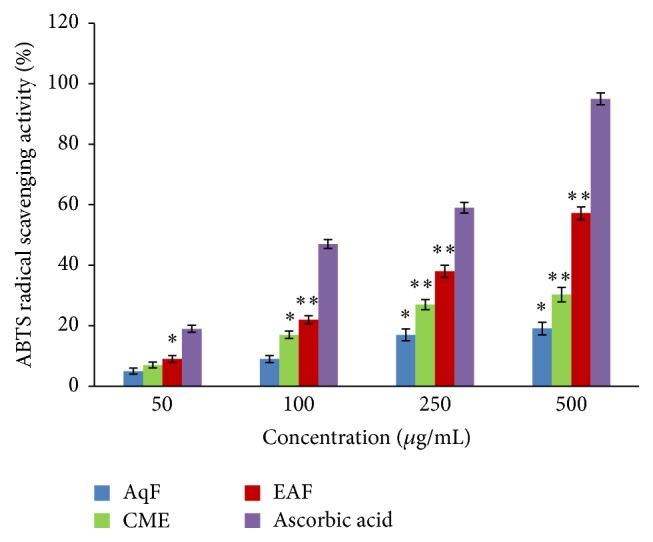
ABTS^•+^ radical-scavenging assay of the extracts and fractions. EDTA was used as the positive control. Each value is expressed as a mean ± SD (*n* = 3). ^*∗*^ *P* < 0.05 and ^*∗∗*^ *P* < 0.01, compared to the standard with extract and fractions (one-way ANOVA followed by Dunnett's test).

The ABTS assay is one of the popular, very sensitive, practical, rapid, stable, and indirect methods to determine the antioxidant activity of compounds or extracts to scavenge free radicals via electron transfer process [67]. ABTS radical cation (ABTS^•+^), soluble in water as well as organic solvents, was produced by the oxidation of 2,2′-azino-bis(3-ethylbenzothiazoline-6-sulphonate) (ABTS) in the presence of potassium persulfate [68]. The formed radical cation is slightly stable, but it instantly converted into a noncolored form of ABTS in the presence of an H atom donor or antioxidant compounds [69]. This method is very similar to the DPPH^•^ radical-scavenging activity. The ABTS radical-scavenging activities of CME, EAF, AqF, and ascorbic acid were in the following order: ascorbic acid > EAF > CME > AqF at a concentration of 500 *μ*g/mL (Figure 2). The ABTS radical-scavenging percentages of 500 *μ*g/mL of ascorbic acid, CME, EAF, and AqF were 94.6%, 30.3%, 57.2%, and 19.1%, respectively. The higher the value of ABTS radical-scavenging percentages the better the antioxidant properties.

### 3.4. *In Vivo* Antioxidant Activity

CCl_4_, as a free radical generator, is widely used as hepatotoxin in the experimental animal. Biotransformation of CCl_4 _in hepatic parenchyma cells by liver cytochrome P450 dependent monooxygenase into trichloromethylperoxyl radical (CCl_3_O_2_^•^) causes peroxidative degradation of lipid membranes of the hepatic cells. This leads to the formation of lipid peroxides, which in turn give up products like MDA. MDA is a cytotoxic product causing the loss of tangibility of cell membranes and damage to hepatic tissues [70]. SOD, CAT, GSH, and GSH-Px are the prime endogenous antioxidant enzymes and are referred to as the first line of defense against the harmful effects of reactive oxygen species (ROS) and free radicals in biological systems during oxidative stress. These enzymes' system converts harmful active oxygen species into nontoxic compounds. Therefore, the increase in these enzymes may provide an effectual protection from the harmful effects of free radicals to cells or organisms [71]. The superoxide anion (O^2−^) is the most common free radical generated in biological system. SOD, commonly known as cytocuprein, is the only enzyme that catalyzes the dismutation of superoxide radicals (O^2−^) into H_2_O_2_ and O_2_. CAT and GSH can catalyze the decomposition of H_2_O_2_ into H_2_O and O_2_ and are also employed to prevent the formation of OH^−^ radicals with abolishing H_2_O_2_ produced by free radicals. GSH-Px is also employed in the detoxification of H_2_O_2 _to H_2_O and reduces lipid hydroperoxides to their corresponding alcohols. Hence, these enzymes protect the cell free radical induced cellular damage [72]. The effects of EAF on several biochemical parameters of oxidative stress were evaluated in liver and kidney homogenates of treated CCl_4 _rats. On the basis of our* in vitro* antioxidant assays, ethyl acetate fraction was chosen as the most potent extract and therefore it was used to estimate the antioxidant activity of* L. coromandelica in vivo*. As shown in Table 4, CCl_4_ intoxicated group produced significant (*P* < 0.05, *P* < 0.01, and *P* < 0.001) decrease in SOD, CAT, GSH, and GSH-Px levels and increases in the MDA level in the liver and kidney, compared with the intoxicated group in a dose-dependent manner. The results obtained in the present study indicated that EAF (400 mg/kg) produced comparable result as standard vitamin E.

## 4. Conclusion

In conclusion, the present study clearly indicated that the innumerable chemical groups (phenolics, flavonoid, and triterpenes) are present in* L. coromandelica*. Among all, EAF had significant antioxidant activity against various antioxidant systems* in vitro*. EAF also exhibited strong antioxidant activity* in vivo* by showing the enhanced activities of antioxidant enzymes (SOD, CAT, and GSH-Px) and decreased the substances for the lipid peroxidation reaction product (MDA) in livers and kidney of rats. The high antioxidant activity exhibited by* L. coromandelica in vitro* and* in vivo* provided justification for the therapeutic use of this plant in folkloric medicine. On the basis of these finding, it could be considered as a potential source of natural antioxidant that impart a major role for the treatment of radical related diseases and age associated diseases. The next step-up should be ensuing to isolate and identify the specific compounds and correlation with its molecular mechanism.

## Figures and Tables

**Table 1:** Phytoconstituents present in *L. coromandelica*.

Extract/fractions	Phytoconstituents								
	Alkaloid	Steroid	Triterpenes	Glycoside	Phenolic	Flavonoid	Tannin	Sugar	Saponins
Methanol	**+**	**+**	**+**	**−**	**+**	**+**	**+**	**−**	**+**
Ethyl acetate	**+**	**+**	**+**	**−**	**+**	**+**	**+**	**−**	**+**
Aqueous	**+**	**−**	**−**	**−**	**+**	**+**	**+**	**−**	**+**

+: present; −: absent.

**Table 2:** Total extractable compounds (TEC), total phenolics contents (TPC), total flavonoid contents, and total antioxidant capacity (TOAC) of methanolic extract/fractions of *L. coromandelica*.

	Crude methanolic extract	Ethyl acetate fraction	Aqueous fraction
	(CME)	(EAF)	(AqF)
Total extractable compounds (TEC) (mg/g)	342.21 ± 0.02	256.7 ± 0.27	798.5 ± 0.19
Total phenolics contents (TPC) (mg GAE/g)	32.15 ± 0.18	48.18 ± 0.09	15.71 ± 0.17
TPC/TEC (%)	9.39	18.76	1.96
Total flavonoid contents (TFC) (mg QE/g)	29.1 ± 0.08	41.4 ± 0.12	13.8 ± 0.24
Total antioxidant capacity (TOAC) (mg AAE/g)	61.6 ± 0.06	88.6 ± 0.04	30.4 ± 0.09

All values were expressed as the mean of triplicates ± standard deviation (SD).

**Table 3:** EC_50_ values in different *in vitro* antioxidant assay methods.

Antioxidant assay methods	Standard	Crude methanolic extract	Ethyl acetate fraction	Aqueous fraction
		(CME)	(EAF)	(AqF)
DPPH (*µ*g/mL)	46.4 ± 0.23	86.5 ± 0.78	63.9 ± 0.64	ND
Reducing power assay (mg/mL)	1.3 ± 0.04	17.1 ± 0.24	8.2 ± 0.12	23.6 ± 1.2
Superoxide anion radical scavenging assay (mg/mL)	4.12 ± 0.18	18.72 ± 0.67	11.9 ± 0.23	23.89 ± 0.92
Ferrous ion-chelating activity (mg/mL)	0.23 ± 0.05	16.2 ± 0.25	6.2 ± 0.09	35.8 ± 0.57

Results were expressed as the averages of triplicates ± standard deviation (SD).

**Table 4:** *In vivo* effects of ethyl acetate extract of *L. coromandelica* on liver and kidney SOD, CAT, MAD, GSH, and GSH-px in CCl_4_ treated rats.

Group	Liver					Kidney				
	SOD	CAT	MAD	GSH	GSH-px	SOD	CAT	MAD	GSH	GSH-px
	(U/mg protein)	(U/mg protein)	(nmol/mg protein)	(U/mg protein)	(U/mg protein)	(U/mg protein)	(U/mg protein)	(nmol/mg protein)	(U/mg protein)	(U/mg protein)
I	307.34 ± 36.4	56.51 ± 8.26	6.44 ± 0.79	4.68 ± 0.74	783.45 ± 48.24	283.21 ± 35.23	38.67 ± 6.56	5.21 ± 1.32	4.24 ± 1.08	678.46 ± 56.72
II	198.54 ± 28.51	29.76 ± 5.34	11.87 ± 1.47	2.73 ± 0.36	319.78 ± 36.82	162.51 ± 28.12	21.36 ± 4.89	8.86 ± 1.08	2.18 ± 0.57	243.76 ± 45.29
III	287.54 ± 28.65^*∗∗∗*^	48.34 ± 3.76^*∗∗∗*^	7.32 ± 0.94^*∗∗∗*^	3.84 ± 0.24^*∗∗∗*^	674.25 ± 39.64^*∗∗∗*^	236.62 ± 18.53^*∗∗*^	33.82 ± 6.61^*∗∗*^	6.08 ± 0.87^*∗∗∗*^	3.69 ± 0.87^*∗∗*^	576.82 ± 29.76^*∗∗∗*^
IV	219.21 ± 31.17	34.22 ± 7.93	10.24 ± 1.32^*∗*^	3.16 ± 0.43	423.38 ± 42.81^*∗∗*^	178.64 ± 32.18	25.92 ± 3.78	7.35 ± 0.93	2.67 ± 0.24	352.34 ± 38.62^*∗∗*^
V	253.42 ± 23.67^*∗∗*^	39.56 ± 3.86	9.34 ± 0.84^*∗∗∗*^	3.45 ± 0.36^*∗*^	512.39 ± 42.54^*∗∗∗*^	202.53 ± 42.13^*∗*^	28.73 ± 5.48	7.06 ± 0.89^*∗*^	2.96 ± 0.38	408.13 ± 24.62^*∗∗∗*^
VI	279.31 ± 19.2^*∗∗∗*^	43.65 ± 9.65^*∗∗*^	8.67 ± 0.47^*∗∗∗*^	3.73 ± 0.21^*∗∗*^	612.75 ± 56.34^*∗∗∗*^	223.86 ± 29.64^*∗∗*^	31.28 ± 4.94^*∗*^	6.84 ± 0.76^*∗∗*^	3.29 ± 0.29^*∗*^	459.31 ± 21.8^*∗∗∗*^

Values are expressed as mean ± SD (*n* = 6) and evaluated by one-way ANOVA. Statistical significance is indicated by asterisks.

^*∗*^ *P* < 0.05, ^*∗∗*^ *P* < 0.01, and ^*∗∗∗*^ *P* < 0.001, compared to CCl_4_ intoxicated group.
